# Myocardial perfusion reserve of kidney transplant patients is well preserved

**DOI:** 10.1186/s13550-020-0606-6

**Published:** 2020-02-10

**Authors:** Johanna Päivärinta, Kaj Metsärinne, Eliisa Löyttyniemi, Jarmo Teuho, Tuula Tolvanen, Juhani Knuuti, Niina Koivuviita

**Affiliations:** 1 Department of Medicine, Division of Nephrology, Turku University Hospital, PL 52, Kiinamyllynkatu 4-8, 20521 Turku, Finland; 2 Turku PET Centre, University of Turku, Turku, Finland; 3 Department of Biostatistics, University of Turku, Turku, Finland; 4 Department of Medicine, University of Turku, Turku, Finland; 5 Department of Medical Physics, Division of Medical Imaging, Turku University Hospital, Turku, Finland

**Keywords:** Renal transplant, Coronary perfusion, Positron emission tomography (PET), Kidney impairment

## Abstract

**Background:**

Chronic kidney disease (CKD) is associated with endothelial dysfunction and increased cardiovascular mortality. Endothelial dysfunction can be studied measuring myocardial perfusion reserve (MPR). MPR is the ratio of stress and rest myocardial perfusion (MP) and reflects the capacity of vascular bed to increase perfusion and microvascular responsiveness. In this pilot study, our aim was to assess MPR of 19 patients with kidney transplant (CKD stages 2–3) and of ten healthy controls with quantitative [^15^O]H_2_O positron emission tomography (PET) method.

**Results:**

Basal MP was statistically significantly higher at rest in the kidney transplant patients than in the healthy controls [1.3 (0.4) ml/min/g and 1.0 (0.2) ml/min/g, respectively, *p* = 0.0015]. After correction of basal MP by cardiac workload [MP_corr_ = basal MP/individual rate pressure product (RPP) × average RPP of the healthy controls], the difference between the groups disappeared [0.9 (0.2)  ml/min/g and 1.0 (0.3) ml/min/g, respectively, *p* = 0.55)]. There was no difference in stress MP between the kidney transplant patients and the healthy subjects [3.8 (1.0) ml/min/g and 4.0 (0.9) ml/min/g, respectively, *p* = 0.53]. Although MPR was reduced, MPR_corr_ (stress MP/basal MP_corr_) did not differ between the kidney transplant patients and the healthy controls [4.1 (1.1) and 4.3 (1.6), respectively, *p* = 0.8].

**Conclusions:**

MP during stress is preserved in kidney transplant patients with CKD stage 2–3. The reduced MPR appears to be explained by increased resting MP. This is likely linked with increased cardiac workload due to sympathetic overactivation in kidney transplant patients.

## Background

The cardiovascular mortality risk is 10–20 times higher in dialysis patients compared to the general population [1]. Although cardiovascular (CV) mortality seems to halt in patients with kidney transplant [2], their outcome still remains worse than that of the general population [3].

Endothelial dysfunction and oxidative stress are associated with rapidly developing atherosclerosis in advanced kidney disease [4]. The amount of nitric oxide (NO) and angiogenesis inhibitors also seem to increase in uremia inducing endothelial to mesenchymal transition in the myocardium. Consequently, fibrosis and capillary rarefaction occur [5, 6].

Myocardial perfusion reserve (MPR), the ratio of hyperemic and basal blood perfusion, integrates the hemodynamic effects of epicardial stenosis, diffuse atherosclerosis, smooth muscle relaxation, and endothelial function [7]. CV mortality is known to increase with declining MPR [8]. In manifest epicardial coronary artery disease (CAD), stress myocardial perfusion (MP) begins to decrease after 40% of coronary stenosis [9]. Decreased MPR has been also documented in several preatherosclerotic states like hypertension [10] and dyslipidemia [11] without obstructive CAD. Furthermore, reduced MPR has been established in patients with mild to severe CKD [12–14], and it is associated with CV mortality also in patients with CKD [15, 16].

There are only a few Doppler echo-based studies of MPR in patients with kidney transplant [17, 18]. Studies of MPR by positron emission tomography (PET) which has been considered the gold standard of quantitative tissue perfusion measurement are lacking in this patient group. Furthermore, there are no prospective studies comparing MPR measured while on dialysis treatment versus after transplantation. In cross-sectional Doppler-based studies, MPR has shown to be better in patients with kidney transplant than in patients in dialysis [17, 18].

In this pilot study, our aim was to assess MPR of kidney transplant patients without manifest atherosclerosis by means of [^15^O]H_2_O PET and evaluate the technique for our future prospective study.

## Methods

### Subjects

Nineteen kidney transplant patients with estimated glomerular filtration rate (eGFR) > 30 ml/min and 10 healthy control subjects were included in the study. Patients were recruited from the nephrology outpatient clinic of Turku University Central Hospital during 2017–2018. Our aim was to study microvascular function. Thus, patients with CKD 4 (eGFR < 30 ml/min) and/or with abdominal calcification score (AAC) > 8 and/or with any clinical signs of atherosclerotic disease (CAD, cerebrovascular disease, peripheral artery disease) were excluded. None of the healthy controls had any history of heart or kidney disease or were on any medication.

### Study design

Basal and stress MP and MPR were measured with [^15^O]H_2_O PET. Laboratory samples were taken at the time of myocardial imaging. Pretransplantation AAC score was assessed from lateral lumbar radiography. Echocardiography was made as pretransplantation examination while the patients were in predialysis follow-up or in dialysis.

### Myocardial PET

The imaging studies were carried out after a 10-h overnight fast. Caffeine and alcohol were prohibited for 1 day before assessment. Patients were instructed to take their medication as usual at study day except angiotensin-converting enzyme (ACE) inhibitors and angiotensin receptor blockers (ARB), which were discontinued 3 days before imaging due to renal imaging on the same day.

Venous catheter was placed in an antecubital vein for injection of oxygen-15-labeled water [^15^O]H_2_O and adenosine. The subjects were positioned supine in the camera (Discovery 690 PET/CT scanner, GE Medical Systems, Waukesha, Wisconsin, USA). A low-dose helical CT scan with automatic dose modulation (120 kVp, 10–80 mAs, noise index 30, pitch of 1.375, rotation time of 0.5 s) was acquired during normal breathing before the resting PET scan to correct for photon scatter and attenuation. Electrocardiogram (ECG), heart rate (HR), and blood pressure were monitored continuously during the studies.

Rest and stress MP imaging with [^15^O]H_2_0 PET was performed as described earlier [19]. Oxygen-15-labeled water (470 MBq) was injected (Radiowater Generator, Hidex Oy, Finland) at rest, and simultaneously, a PET perfusion scan was started. The dynamic acquisition scan of 4 min 40 s was performed with corresponding frame times of (14 × 5 s, 3 × 10 s, 3 × 20 s, and 4 × 30 s). After a 10-min decay of the [^15^O]H_2_O radioactivity, an identical [^15^O]H_2_O PET (470 MBq) sequence was performed during hyperemia. Adenosine was initiated 2 min before the stress scan for maximal vasodilatation. The mean radiation dose was 1 mSv for the perfusion study.

The PET data were reconstructed using 3D ordered subset expectation maximization (vendor name: VUE Point HD) with point spread function modelling (vendor name: SharpIR) with a 128 × 128 matrix and FOV of 350 mm in size. The reconstructions were performed using 2 iterations, 24 subsets, and a Gaussian post-filter of 6.4 mm. The PET images were reconstructed with all quantitative corrections applied to the reconstructed images, including attenuation, scatter, decay, and random corrections.

Global MP was analyzed with Carimas software [19, 20]. Arterial input function was extracted directly from the dynamic PET data. Single-tissue compartment model was used with correction for perfusable tissue fraction to generate parametric MP images [19, 20]. MP was expressed in milliliters per minute per gram of perfusable tissue (ml/min/g).

### Calculation formulas of MP

MPR was calculated as the ratio of stress-to-rest MP. Because basal MP is related to the rate pressure product (RPP), an index of myocardial oxygen consumption, basal MP values were corrected for RPP (systolic blood pressure × HR) by the equation: basal MP_corr_ = basal MP/individual RPP × average RPP of the healthy controls [21]. The average RPP of the healthy was used to make comparison of perfusion values between the patients and the healthy controls easier. Corrected MPR (MPR_corr_) was defined as the ratio of hyperemic MP divided by basal MP_corr_. Coronary vascular resistance (CVR) was calculated as mean arterial pressure (MAP) divided by global MP.

MAP after adenosine administration was calculated as mean of the 3- and 6-min MAP. Stress MP > 2.3 ml/min/g, and MPR > 2.5 were considered normal based on previous validation [19].

### Echocardiography

Echocardiography was performed in 18/19 patients as pretransplantation examination while the patients were in predialysis follow-up or in dialysis. The time between PET imaging and echocardiography varied from 1 year to 7 years. Left ventricular mass index (LVMI) and ejection fraction (EF) were measured. LVMI calculation was based on the Devereux equation [22] and normal values of LVMI on American Society of Echocardiography (ASE) convention [23]. According to ASE, reference range of LVMI for men is 49–115 g/m^2^ and for female 43–95 g/m^2^.

### AAC score

The individual risk of atherosclerosis was evaluated before transplantation by AAC score in lateral lumbar radiography in 17/19 patients [24]. The time between PET imaging and AAC score varied from 1 year to 7 years. We excluded patients with AAC score of more than 8/24.

### Assessment of renal function

The assessment of renal function was based on the eGFR equation from The Chronic Kidney Disease Epidemiology Collaboration (CKD-EPI) study [25]. CKD was referred according to the KDOQI definition. S-Crea and eGFR were measured within one month of PET imaging.

### Statistical analysis

Comparisons between healthy controls and kidney transplant patients for continuous parameters were performed with Kruskal–Wallis test. Additional analyses were performed when males and females, diabetic and non-diabetic, were compared. In addition, correlation coefficients were calculated when associations were examined. All statistical tests were performed as two tailed, with a significance level set at 0.05. The analyses were performed using SAS System, version 9.4 for Windows (SAS Institute Inc., Cary, NC, USA).

## Results

### Study subjects

The demographics of the study subjects are shown in the Table 1. Causes of CKD were as follows: 6 IgA nephropathies, 4 type I diabetic nephropathies, 1 lupus nephritis, 4 autosomal dominant polycystic kidney diseases, 2 medullary cystic kidney diseases, 1 FSGS, and 1 kidney disease without a specific diagnosis. One of the patients had a kidney-pancreas transplantation. Five patients were in hemodialysis (HD) before kidney transplantation and 14 patients in peritoneal dialysis (PD).

**Table 1:** Baseline characteristics

	Kidney transplant patients (*N* = 19)	Controls (*N* = 10)
Age (years)	52 (23–70)	56 (48–64)
BMI (kg/m2)	28 (5)	26 (4)
Sex F/M (N)	10/9	7/3
eGFR (ml/min)	57 (13)*	83 (6)
P-Crea (μmol/l)	115 (24)*	75 (11)
P-ProBNP (ng/l)	199 (191)*	119 (121)
P-TnT (ng/l)	10 (6)*	5 (0)
fP-chol (mmol/l)	4.9 (1.0)	
fP-LDL (mmol/l)	2.8 (0.8)	
fP-HDL (mmol/l)	1.5 (0.4)	
fP-Tg (mmol/l)	1.6 (0.9)	
B-Hb (g/l)	140 (11)	
fP-gluk (mmol/l)	6.0 (1.7)	
U-prot (g/l)	0.1(0.4)	
Hypertensio arterialis (N)	19	0
DM I/II (N)	4/0	0
Smoking (N)	0	0
Time in dialysis (months)	21 (14)	0
Age of kidney transplant (months)	37 (23)	

Values are mean (SD)

*BMI* body mass index

*p < 0.05 controls versus kidney transplant patients

All the kidney transplant patients were on antihypertensive medication. Seven of 19 patients had either ACE inhibitor or ARB. Calcium channel blocker was used by 16 patients, beta blocker by 15, and diuretic by 8. There were 4 patients who had a combination of 3 antihypertensives and 3 who had a combination of 4 antihypertensives. Statins were used by 11 patients.

Four patients used a combination of tacrolimus, mycophenolate, and corticosteroid as immunosuppressive medication; seven patients used a combination of cyclosporine, mycophenolate, and corticosteroid; a combination of cyclosporine and mycophenolate was used by four patients and a combination of tacrolimus and mycophenolate by four patients.

AAC score was 0 in 13 patients, 3 in one, 5 in one, 6 in one, and 8 in one patient, and two of the patients did not have AAC score measured at all. Echocardiography was performed in 18/19 patients and there was an increased LVMI in 8/18 patients [mean LVMI 92 (26)]. All patients had normal EF [mean EF 66 (5)%].

There was a statistically significant difference in eGFR and plasma creatinine between controls and transplant patients (*p* < 0.0001). P-ProBNP and P-Tnt were significantly higher in the kidney transplant group than in the controls (*p* = 0.013 and *p* = 0.0009, respectively).

### Hemodynamic measurements during imaging

Hemodynamic measurements are shown in Table 2. Systolic and diastolic blood pressure, MAP, and HR at rest were higher in the transplant group than in the healthy controls (*p* = 0.0023, *p* = 0.023, *p* = 0.0044, and *p* = 0.0083, respectively). Systolic blood pressure and MAP at stress were higher in the kidney transplant patients than in the healthy controls (*p* = 0.03 and 0.04, respectively).

**Table 2:** Blood pressure and heart rate of the study subjects

	Kidney transplant patients (*N* = 19)	Controls (*N* = 10)
Rest		
BP systolic (mmHg)	151 (21)*	123 (16)
BP diastolic (mmHg)	82 (16)*	70 (9)
MAP (mmHg)	106 (16)*	88 (11)
HR (beats/min)	66 (12)*	55 (4)
Stress		
BP systolic (mmHg)	138 (17)*	120 (18)
BP diastolic (mmHg)	71 (8)	65 (9)
MAP (mmHg)	93 (9)*	84 (11)
HR (beats/min)	89 (13)	86 (14)

Values are mean (SD)

*BP* blood pressure, *MAP* mean arterial pressure, *HR* heart rate

*p < 0.05 controls versus kidney transplant patients

### Myocardial perfusion

MP values are presented in Table 3. Basal MP as well as RPP were statistically significantly higher in the kidney transplant patients than in the control subjects (*p* = 0.0044 and *p* = 0.0015, respectively). The difference of basal MP between the groups disappeared after correction of basal MP by RPP (*p* = 0.31). There was no difference of statistical significance in stress MP or stress MPR between the groups (*p* = 0.53; *p* = 0.15, respectively). There were no differences in regional stress MP between the main coronary arteries. MPR in kidney transplant patients was lower than in controls, and the difference was statistically significant (*p* = 0.0029). However, after correction by RPP, the difference between the groups disappeared (*p* = 0.8).

**Table 3:** Myocardial flow values in all subjects

	Kidney transplant patients *N* = 19	Controls *N* = 10
Basal MP (ml/min/g)	1.3 (0.4)*	1.0 (0.2)
RPP	10,053 (2878)*	6723 (1112)
Basal MP_corr_ (ml/min/g)	0.9 (0.2)	1.0 (0.3)
Stress MP (ml/min/g)	3.8 (1.0)	4.0 (0.9)
MPR	3.0 (0.9)*	4.2 (1.0)
MPR_corr_	4.3 (1.6)	4.1 (1.1)
CVR_basal_ (mmHg mL^-1^ min^-1^ g^-1^)	83 (19)	93 (21)
CVR_stress_ (mmHg mL^-1^ min^-1^ g^-1^)	27 (9)	22 (8)

Values are mean (SD)

*RPP* rate pressure product, *Basal MP*_*corr*_ corrected basal MP, MP/individual RPP × RPP average of the healthy, *MPR* myocardial perfusion reserve, *MPR*_*corr*_ corrected myocardial perfusion reserve, stress MP/basal MP_corr)_, *CVR* coronary vascular resistance

*p < 0.05 controls versus kidney transplant

### The effect of different parameters on myocardial perfusion

There was no statistically significant difference in MP values between diabetics and non-diabetics (basal MP, *p* = 0.74; stress MP, *p* = 0.19; MPR, *p* = 0.31) or between women and men (basal MP, *p* = 0.11; stress MP, *p* = 0.66; MPR, *p* = 0.14). The previous dialysis type did not have any statistically significant effect on MP (basal MP, *p* = 0.28; stress MP, *p* = 0.26; MPR, *p* = 0.85). There was no statistically significant correlation between Hb, subject’s age, age of the transplant, dialysis vintage, BMI or EF and basal or stress MP or MPR (*p* > 0.05 in all). There was a statistically significant correlation between LVMI and stress MP and MPR_corr_ (*r* = 0.54, *p* = 0.02; *r* = 0.69, *p* = 0.0017, respectively).

Basal MP correlated with eGFR when 29 subjects of both groups were combined (*r* = − 0.43, *p* = 0.019). However, the correlation disappeared after correction by cardiac workload (*r* = − 0.08, *p* = 0.69). There was no correlation of statistical significance between the change of eGFR after transplantation (difference between eGFR at 1 year after transplantation and at the time of PET imaging) and MP values (basal MP, *p* = 0.42; stress MP, *p* = 0.63; MPR, *p* = 0.76).

## Discussion

This is the first study to report MP values of patients with kidney transplant based on [^15^O]H_2_O PET which is considered the gold standard method of measuring tissue perfusion. The main finding of this study was that MP and CVR during stress are preserved in the kidney transplant patients with CKD stage 2–3. The reduced MPR appears to be explained by increased resting MP. This is likely linked with increased cardiac workload in transplant patients.

Basal MP was elevated 1.3 (0.4) ml/min/g in the kidney transplant patients compared to the value 1.0 (0.2) ml/min/g of the healthy controls. Because the resting MP is related to myocardial work and metabolic demand, HR, and systolic blood pressure must be considered when comparing MP basal values within a study [21]. After RPP correction, the basal MP of the kidney transplant patients was equal with the MP of the healthy controls. Consequently, MPR was also equal in both groups after correction by cardiac workload.

Elevated RPP in patients with kidney transplant can be explained by sympathetic overactivation. Sympathetic overactivation has been established already in early stages of CKD [26]. Studies of autonomic nervous system in kidney transplant patients, based on HR variability and muscle sympathetic nerve activity, have shown that dysfunction of autonomic nervous system may improve after transplantation but it may also persist [27, 28].

Stress MP did not differ between the groups. It is very likely, that there was no obstructive CAD either in the patients or in the healthy controls, because there were no regional differences in stress MP between main coronary arteries. Furthermore, we used pretransplantation AAC score to estimate CV risk. Increasing AAC score, especially score values greater than 8–15, has been associated with severely increased risk for CV events in dialysis patients [29–31]. AAC score 8 has been used as a cut-off value for high calcification in transplant patients [32, 33]. Lewis et al. showed, that there is a continuous 7–8% increase in risk of CV events for each 1 point increase in AAC score without an exact cut-off point [33]. Based on these previous studies, AAC-score 0 in 13/17 patients and the highest AAC score 8/24 in our study should indicate mild to moderate CV risk.

### Myocardial perfusion in patients with CKD

Like in our study, Charytan et al. did not find a statistically significant difference in stress MP and MPR by means of [^13^N] ammonia PET between patients with stages 1–3 CKD and the healthy controls [34]. Similarly, a previous study of our group (Koivuviita et al.) showed by means of [^15^O]H_2_O PET comparable stress MP between patients with stages 3–5 CKD and the control subjects [35]. Fukushima et al. had a finding pointing to the same direction in a [^82^Rb] PET study in patients with CKD stage 3 [13]. Furthermore, in an intracoronary guidewire study of Chade et al. with patients with CKD 3 without obstructive CAD, there was no difference in stress MP compared to the healthy controls [12].

Decreased stress MP has been demonstrated in patients with dialysis-dependent CKD [14, 17, 18, 36]. It is possible, that repeated dialysis sessions cause microvascular trauma decreasing stress MP. In our study, the average dialysis vintage was 21 months which was clearly shorter when compared with above-mentioned studies (48–95 months) [14, 17, 18, 36]. Accordingly, a correlation between dialysis vintage and MPR was shown in dialysis patients in two Doppler studies [17, 18].

### Myocardial perfusion in patients with kidney transplant

In contrast to our study, decreased stress MP has been reported in patients with renal allograft with mild kidney impairment by means of Doppler [17, 18, 37]. Obstructive CAD was excluded on a clinical basis in those studies. The length of dialysis time before transplantation was shorter in our study (21 months) than in the Doppler-based studies (39–64 months) [17, 18, 37], which may have had impact on different results. In concordance with that assumption, there was a negative correlation between MPR and previous dialysis vintage of transplant patients in Doppler studies [17, 18]. We did not find any correlation between dialysis vintage and MPR, perhaps based on the short durance of dialysis treatment of our patients.

LVH has been associated with both decreased and increased stress MP [38, 39]. In the above-mentioned Doppler studies, LVH was highly prevalent in transplant patients [17, 18]. In our patients, there was increased LVMI in 8 of 18 patients in Doppler echo before transplantation. However, prevalence of increased LVMI at the time of PET scan was not known. There was a positive correlation between LVMI and stress MP in our study. In contrast to our result, LVMI correlated negatively with stress MP in Doppler studies [17, 18].

### Limitations

There are some limitations in our study. Due to the long interval between echocardiography and PET imaging LVMI changes cannot be excluded [40]. However, there were no manifest heart failure episodes between the examinations probably speaking for heart failure with preserved EF.

In addition, the impact of antihypertensive medication on our results cannot be excluded, because only ATR blockers and ACE inhibitors were discontinued before the imaging. However, RAAS (renin-angiotensin-aldosterone-system) blockage has been most strongly associated with increased MPR [41, 42]. Finally, the sample size of our study was quite small.

## Conclusion

In conclusion, this study showed the capability of [^15^O]H_2_O PET in measuring MP of patients with kidney transplant. The difference in MPR between the healthy controls and the patients with kidney transplant can be explained by increased cardiac workload in transplant patients, which is probably associated with increased sympathetic activity. We are continuing our cardiac studies with prospective cohort of dialysis patients on kidney transplant waiting list.
